# Walking as an intervention to reduce blood pressure in adults with hypertension: recommendations and implications for clinical practise

**DOI:** 10.12968/bjca.2022.0119

**Published:** 2023-03-20

**Authors:** Mohamed Mansoor, Oliver Hamer, Kirishan Sandrasekar, Candiss Argent, James Hill

**Affiliations:** 1 NHS Blackpool Teaching Hospitals NHS Foundation Trust, Lancashire, UK; 2 NIHR Applied Research Collaboration - Northwest Coast, University of Central Lancashire, Preston, UK; 3 Lancashire Teaching Hospitals NHS Foundation Trust, Preston, UK

**Keywords:** Hypertension, Physical activity, Trials, Blood pressure, Systematic review

## Abstract

Hypertension affects more than 1 in 4 adults, equating to around 1.39 billion people worldwide. Hypertension is defined as an elevation in systolic blood pressure above 140mmHg. This can increase cardiovascular and stroke risk. By lowering blood pressure, individuals can mitigate an increased risk of stroke and end-organ damage. While medications have proven beneficial to lowering blood pressure, they do have the potential for side effects. Other non-pharmacological lifestyle and dietary changes exist. This article critically appraises a systematic review which assesses whether walking can reduce blood pressure.

## Introduction

Hypertension is defined as elevated blood pressure above 140mmHg (systolic) (1). This can potentiate risks to the heart, the brain (in the form of strokes), and organ damage (through peripheral vascular disease) (2). By maintaining normal blood pressure within acceptable parameters, it consequently lowers cardiovascular risk (3). Lowering this risk is important, especially given that it remains the largest risk factor of cardiovascular disease (1). In the UK alone, elevated blood pressure is the third biggest risk for disease after diet and tobacco (4).

Hypertension affects more than 1 in 4 adults equating to around 1.39 billion people worldwide (5). However, a high number of people that have high blood pressure are not always aware of it (often asymptomatic) (6). The effects of undiagnosed hypertension not only increase morbidity and mortality, but places increased demands on healthcare systems by prolonging hospital stays and increased follow up care (6).

Lowering blood pressure can be achieved in multiple ways, with medications and by making conscious lifestyle changes (2). Lifestyle changes can be in the form of eating a balanced, low salt diet, reducing cigarette smoking and increasing physical activity (7, 8). Studies have suggested that physical activity is one of the most effective interventions for significantly lowering blood pressure (9, 10). One of the more popular activity interventions for adults with hypertension is that of walking (11). These interventions have become more popular in recent years because they come at little cost, are very accessible, flexible with people’s lifestyle, and are independent of any equipment (12). A recent Cochrane systematic review by Lee et al (2022) aimed to determine the effect of walking as a physical activity intervention on blood pressure and heart rate (11).

### Aim of commentary

This commentary aims to critically appraise the methods used within the Cochrane systematic review Lee et al, (2021) and expand upon the findings in the context of clinical practice (11).

### Methods of the review by Lee et al, 2021

Fourteen databases were searched: Cochrane Register of Studies; MEDLINE, Embase Ovid, CINAHL EBSCO, SPORTDiscus EBSCO, PsychINFO EBSCO, Physiotherapy Evidence Database (PEDro); ClinicalTrials.gov; World Health Organisation International Clinical Trials Registry Platform; Index of Taiwan Periodical Literature System; National Digital Library of Theses and Dissertations in Taiwan; China National Knowledge Infrastructure − Journals, Theses & Dissertations; Wanfang Medical Online. The searches were conducted from inception of database to March 2020, with no restriction on languages or publication status.

Only individually randomised parallel group-controlled trials with adults who were hypertensive and normotensive (16 years and over) were included. The comparisons were non-exercising and non-intervention groups. All trials included walking interventions in the community or laboratories. Trials with mixed interventions such as walking, and jogging were excluded. The primary outcome measure was systolic blood pressure. Diastolic blood pressure and heart rate were secondary outcome measures (11). Screening, data extraction and assessment of bias (Cochrane ‘Risk of Bias’ tool and The Grading of Recommendations Assessment, Development and Evaluation GRADE) was undertaken by two reviewers independently. Any discrepancies were mediated with a third reviewer. Where appropriate random effects meta-analysis was employed to analyse the data. The pooled results of systolic and diastolic blood pressure and heart rate were calculated and presented as mean differences between groups with confidence intervals of 95%. To assess for heterogeneity between the results of each studies, forest plots, Chi^2^ tests and I^2^ were utilised. Subgroup analyses were conducted for age and sex where the studies had sufficient information. A sensitivity analysis was undertaken to assess the effect of including high/low and low risk of bias studies.

## Results

The Cochrane systematic review included 73 randomised control trials involving 6473 participants. Trials were conducted across 22 countries, mainly in the USA, UK, China, Canada, and Taiwan. The age of participants ranged between 16 to 84 years with just over half (51%) over 60 years of age. Among the total sample, 39% of participants were aged 41 to 60 years with the remaining 10% being 40 years and younger. Trials largely recruited both males and females (n= 49), however four trials only recruited males. In total, there were 1.5 times as many female participants compared to male participants (3122 versus 2075). Interventions were carried out in the home, in the community, or in a laboratory (e.g., treadmills). The average length of interventions across all studies was 15 weeks, consisting of three to five sessions per week (20 to 40 minutes in duration). Participants in the control groups received no intervention (11).

### Effectiveness of walking Intervention versus no intervention on blood pressure and heart rate

#### Outcome 1: Blood pressure

Meta-analysis showed that walking reduced systolic blood pressure (SBP) and diastolic blood pressure (DBP) compared to no intervention in hypertensive and normotensive adults aged 16 years and over (MD -4.11 mmHg, 95% CI -5.22 to -3.01: moderate-certainty evidence; and MD -1.79 mmHg, 95% CI -2.51 to -1.07: low certainty evidence, respectively).

#### Outcome 1: Sub-group analysis

In participants under 40 years of age, meta-analysis showed that walking lowered systolic blood pressure and diastolic blood pressure compared to no intervention (MD of -4.41 mmHg, 95% CI -6.17 to -2.65, moderate certainty evidence; and MD of -3.01 mmHg, 95% CI -4.44 to -1.58: Moderate certainty evidence, respectively).

In participants aged 41 to 60 years, meta-analysis showed that walking lowered systolic blood pressure and diastolic blood pressure compared to no intervention (MD of -3.79 mmHg, 95% CI -5.64 to -1.94: low certainty evidence; and MD of -1.74 mmHg, 95% CI -2.95 to -0.52: Low certainty evidence, respectively).

In participants aged 60 years or over, meta-analysis showed that walking lowered systolic blood pressure and diastolic blood pressure compared to no intervention (MD of -4.30 mmHg, 95% CI -6.17 to -2.44: Low certainty evidence; and MD of -1.33 mmHg, 95% CI -2.40 to -0.26: Low certainty evidence, respectively).

In female and male participants, meta-analysis showed that walking lowered systolic blood pressure compared to no intervention (MD -5.65 mmHg, 95% CI -7.89 to -3.41: Low certainty evidence; and MD -4.64 mmHg, 95% CI -8.69 to -0.59: Low certainty evidence, respectively).

In female and male participants, meta-analysis showed that walking lowered diastolic blood pressure compared to no intervention (MD -2.69 mmHg, 95% CI -4.16 to -1.23: Low certainty evidence; and MD -2.54 mmHg, 95% CI -4.84 to -0.24: Moderate certainty evidence, respectively)

When only calculating trails with significant reduction in systolic blood pressure, the average walking duration per week was approximately 151 minutes (range 60 − 220mins). Similarly, moderate intensity walking was the major prescription in trials with a significant reduction in systolic blood pressure.

#### Outcome 2: Heart rate

Meta analysis showed that walking significantly reduced heart rate (HR) compared to no intervention in hypertensive and normotensive adults aged 16 years and over (MD -2.76 bpm, 95% CI -4.57 to - 0.95: low certainty evidence).

#### Outcome 3: Adverse events

Of the included studies, 21 trials reported a total of eight adverse events. Adverse events included stress fracture, knee injury, knee pain, bruised foot, and acute cholecystitis. Knee pain was the most common adverse event reported by participants within the intervention groups (n = 4).

## Commentary

Using the AMSTAR 2 critical appraisal tool for systematic reviews, all 16 criteria were judged to be satisfactory for this review (table 1) (13). The Cochrane systematic review comprehensively detailed the relevant PICO, detailed the process of study selection, and data extraction. Both study selection and data extraction were conducted independently by two authors. The review included a comprehensive literature search, not including grey literature search because of the initial inclusion criteria of RCT. The review authors conducted an appropriate meta-analysis, risk of bias assessment, investigation of heterogeneity and publication bias. Overall, the systematic review provides a comprehensive synthesis of the included studies (11).

In terms of clinical practise, the findings from this review identified that walking interventions of 20-40 mins, 3-5 times per week for around 15 weeks (in any environment; outdoors or indoors), significantly reduced blood pressure and heart rate (in adults 16 years and over).The evidence suggests that under 40’s had the greatest effect of reduced blood pressure, but the results were relevant for all ages. Furthermore, females appeared to have a greater reduction in blood pressure compared to males.

For clinical practise, this intervention should be encouraged given that adverse events pose less of a risk compared to traditional pharmacological interventions. To support the implementation of walking interventions into clinical practise, the UK Government and WHO both recommend moderate intensity exercise of at least 150-300 minutes per week (14) (5). Although the evidence is consistent with the recommendations, the moderate-low certainty evidence indicates there is some uncertainty about its true level of effectiveness (11). That said, walking has other health benefits including improving cardiovascular health, reducing cardiovascular disease, improving bone health and weight management which provides a further rationale for implementation (14–16). The proven physical benefits aid in blood pressure management (14). Importantly though, walking is yet to be proven to replace any such pharmacological treatments and this should be stressed to patients in the clinical setting. For untrained individuals, exercise should be within the limits of the patient to avoid stressing the cardiovascular system too early on (gradually increasing over time) (10). Based upon the studies within the review, a target frequency and duration of somewhere between 3 to 5 times a week of 20 and 40 minutes should be recommended (11). The majority of studies included within the review used a moderate intensity. To reach moderate intensity of physical activity a recommendation of around a hundred steps per minute can be advised (17). In regard to the environment for walking the studies included in the review were undertaken in a wide range of environments (city/nature/campus). Therefore, when recommending walking environment this should be dependent on patient preference and access. Similarly, regarding the type of walking the included studies in the review varied substantially from treadmill walking, outdoor walking, and nordic walking. Therefore, this may be also decided on patient preference and access.

Future research should focus on reducing bias through high quality randomized control trials which focus particularly on allocation of concealment and blinding outcome assessment. In terms of clinical practice, research should focus on walking at different stages of hypertension. Additionally, future research would focus on adverse events as a primary outcome to establish a safety profile of walking interventions particularly outside versus inside environments (only reported on 21 out of 73 studies) (11). There is currently a lack of cost-benefit analysis of walking interventions versus pharmacological interventions on hypertension. It would be important to characterize the difference in blood pressure reduction between low, moderate, and high intensity exercise. Finally, the authors note that normotensive and hypertensive patients had similar reductions in blood pressure (11). It would be important to further clarify the effect of walking on normotensive versus hypertensive patients (11).

## CPD reflective questions

1. What are the limitations and strengths of the systematic reviews?
2. What are the practical considerations when establishing physical activity guidelines for patients with hypertension?
3. Why is the GRADE approach in systematic reviews used and what are its benefits?

## Figures and Tables

**Table 1:** Critical appraisal using the AMSTAR-2 tool for assessing systematic reviews

AMSTAR-2 questions	Responses
1. Did the research questions and inclusion criteria for the review include the components of PICO?	Yes − The study included all components of PICO
2. Did the report of the review contain an explicit statement that the review methods were established prior to the conduct of the review and did the report justify any significant deviations from the protocol?	Yes − The study reported a protocol with no significant deviations
3. Did the review authors explain their selection of the study designs for inclusion in the review?	Yes - The study explained why they’re not using cluster randomized studies and cross-over trials
4. Did the review authors use a comprehensive literature search strategy?	Yes − A comprehensive search strategy was included.
5. Did the review authors perform the study selection in duplicate?.	Yes - Two reviewers selected a sample of eligible studies and achieved good agreement. Any disagreements were reviewed by a third author.
6. Did the review authors perform data extraction in duplicate?	Yes - At least two reviewers achieved consensus on which data to extract from included studies
7. Did the review authors provide a list of excluded studies and justify the exclusions?	Yes - Included a list of all excluded study and justified the exclusion
8. Did the review authors describe the included studies in adequate details?	Yes - Each included paper incorporated adequate detail
9. Did the review authors use a satisfactory technique for assessing the risk of bias in the individual studies that were included in the review?	Yes - Review authors used a risk of bias tool which included all of the domains
10. Did the review authors report on the sources of funding for the studies included in the review?	Yes -Individual studies included source of funding
11. If meta-analysis was performed did the review authors use appropriate methods for statistical combination of results?	Yes - Authors used random effects model and Chi^2^ and I^2^ values for meta-analysis heterogeneity.
12. If meta-analysis was performed did the review authors assess the potential impact of RoB in individual studies on the results of the meta-analysis or other evidence synthesis?	Yes - Pooled estimates were based on the studies and an analysis was performed on possible impact of the bias.
13. Did the review authors account for RoB in individual studies when interpreting/discussing the results of the review?	Yes - When there was moderate to high risk of bias review included discussion on impact and, conducted a GRADE assessment on certainty of evidence.
14. Did the review authors provide a satisfactory explanation for and discussion of, any heterogeneity observed in the results of the review?	Yes - Where heterogeneity exists, the authors provided an investigation for sources of heterogeneity. The main investigation was performed between subgroups and impact was assessed.
15. If they performed quantitative synthesis did the review authors carry out an adequate investigation of publication bias (small study bias) and discuss its likely impact on the results of the review?	Yes - Publication bias was included in the GRADE assessment. Certainty of evidence was downgraded where publication bias was substantial. Publication bias was also evaluated using funnel plots
16. Did the review authors report any potential sources of conflict of interest, including any funding they received for conducting the review?	Yes - The authors reported no competing interests.
